# LCA applied to comparative environmental evaluation of aggregate production from recycled waste materials and virgin sources

**DOI:** 10.1007/s11356-024-33868-9

**Published:** 2024-06-26

**Authors:** Rafael Linares, Antonio López-Uceda, Andrea Piccinali, Cristina Martínez-Ruedas, Adela P. Galvín

**Affiliations:** 1 Department of Rural Engineering, Civil Constructions and Engineering Projects, University of Córdoba, Andalucía, 14001 Córdoba, Spain; 2 Department of Mechanics, University of Córdoba, Andalucía, 14001 Córdoba, Spain; 3 Department of Civil Engineering, Architecture, Land, Environment and Mathematics, University of Brescia, 25123 Brescia, Italy; 4 Department of Electronic and Computer Engineering, Campus de Rabanales, University of Cordoba, 14071 Córdoba, Spain

**Keywords:** Sustainable construction, Environmental risk impact, Construction and demolition waste, Lifecycle assessment, Recycled aggregate

## Abstract

Nowadays, all productive sectors, including the construction industry, are facing the challenge of reducing their environmental impact. To achieve this objective, numerous actions are being carried out to access greater levels of environmental and economic sustainability. Techniques as Life Cycle Assessment contribute to quantifying environmental impacts, promoting a circular economy in a sector that consumes a high volume of resources, materials, and energy while generating large amounts of gaseous, liquid, or solid emissions. The present study aims to deepen our understanding of aspects that demonstrate the benefits of using RA instead of natural aggregates. This study not only quantifies the environmental impact but also explores the effects of potential improvements in the productive system and their impact on reducing environmental harm. The Life Cycle Assessment methodology is applied to quantify and compare the environmental impacts generated in the production of a ton of mixed recycled aggregates (MRA) from construction and demolition wastes, based on the data provided by plant managers. This is compared to the environmental impacts generated in the production of one ton of natural aggregates extracted from a quarry. The results revealed that the production of mixed recycled aggregate is more environmentally beneficial, confirming a reduction of 70.66% in environmental impacts during the production of recycled aggregates, in comparison to the natural aggregates extraction. Furthermore, the economic analysis demonstrates the economic advantage since the cost of producing recycled aggregates is over 30% cheaper than natural aggregates, being more competitive even when the transportation distances from the plant to the work sites exceed those of natural aggregates.

**Graphical Abstract:**

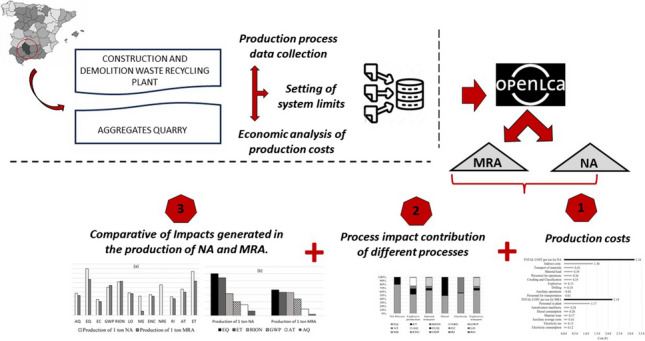

## Introduction

In 2020, the European Union produced 2.5 billion tonnes of waste, with construction and demolition waste (CDW) accounting for 32%, equivalent to 800 million tonnes (Eurostat 2020b). In terms of volume, CDW represents the largest category of waste generated by EU member countries, constituting one-third of all European waste production. This data underscores the significance of proper waste management and increasing recycling rates in promoting sustainability and improving the quality of life (Padilla et al. 2018).

The Waste Framework Directive (WFD, Directive 2008/98/EC updated by Directive (EU) 2018/851) sets a target of achieving a 70% CDW recycling rate by 2020 (Villoria Sáez and Osmani 2019). This, along with the Circular Economy Package and the Circular Economy Action Plan, aimed at facilitating Europe’s transition to a circular economy, emphasizes the importance of secondary raw materials and maintaining the value of waste through reuse and recycling as essential components of a successful circular economy (Padilla et al. 2018). In terms of the recovery rate of mineral waste from CDW, data from the European Environmental Agency (European Union 2022) revealed that the Netherlands led with a recycling rate of 99.8%, followed by Slovenia, Italy, and the United Kingdom with rates of 95–97%, while Denmark, Belgium, Germany, and Greece exceed 80%. Spain and France also achieved rates exceeding 70%.

Previous technical research works focused on unbound RA applications highlighting the feasibility of its use in civil projects such as to fill embankments (Galvín et al. 2018; Liu et al. 2022; Vieira and Pereira 2018) and as material for base or subbase layers on road construction (Agrela et al. 2012; Sarella et al. 2022) or on unpaved roads (Galvín et al. 2013; Huber et al. 2020). Also, the use of RA in the manufacture of recycled construction materials has been evaluated by Silva et al. (2019), Yu et al. (2021), and Lin et al. (2023), which highlights the technical viability of using RA in a broad range of construction applications. As a background review of the present work and the feasibility of using this type of AR from CDW in engineering infrastructures, real-scale studies can be cited as Del Rey et al. (2016a and 2016b) and Galvín et al. (2018). These scientific studies corroborate the high viability that these materials present as construction or filling materials. The most limiting factor in the potential use of RA from CDW recycling plants is the deficiency in the standards/specifications requested for the material produced without comprehensive clauses for the material use. It limits the use of recycled products, which is also due to the low stakeholder’s confidence of its feasibility in real scale applications (De Brito and Silva 2016).

These engineering applications for RA from CDW have been supported by studies focused on their pollutant potential that have confirmed its viability of use from an environmental point of view (Diotti et al. 2020; Galvín et al. 2014, 2018). Furthermore, as an additional application in engineering field, previous research has proven the feasibility of using recycled concrete using this type of waste (Torgal and Ding 2013; De Andrade Salgado and de Andrade Silva 2022). The importance of using RA in concrete has been demonstrated with numerous studies (De Brito, and Agrela, F. (Eds.). 2018; Danish and Mosaberpanah 2022). In this field, experimental studies have demonstrated the feasibility of using this type of aggregates in concrete for real applications. Ulucan et al. (2023) evaluate the construction of a bridge built with natural aggregate concrete in terms of mechanical, environmental, and economic properties, using different ratios of recycled concrete aggregate (RCA). Therefore, the prospect of using green concretes produced using supplementary cementitious material and recycled concrete aggregates in the engineering sector has been widely tested. Research studies such as Ulucan and Alyamac (2023) have evaluated not only its mechanical properties, but also environmental factors such as energy consumption, global warming potential, and waste generation, which have confirmed the high potential for use of RA in concrete manufacturing.

Hence, the advantages of using RA are wide and varied and they can be summarized as follows: (1) It preserves natural resources; (2) it minimizes the emission of CO_2_ into the air and the costs of construction materials, especially for short-distance transportation scenarios; (3) it preserves lands instead of landfilling them and decreases the need for new landfills; and (4) it offers more employment opportunities in the recycling industry (Koppala et al. 2018).

Regarding Spain’s CDW production, approximately 32 million tons were generated in 2020 (Eurostat 2020a). At regional level, the latest available data on 2021 from waste managers and producers, Andalusia, Autonomous Region in southern Spain, generates 1.00 ton of CDW per habitant and year, being the 70% of the CDW generated re-valued (PIREC 2030). In that framework, PIREC (2030), a plan in Andalusia by Junta de Andalucía (2021), outlines strategies to increase CDW recovery, enhance the region’s infrastructure network, support technological innovation in waste treatments, and expand information on CDW recycling and disposal facilities in Andalusia.

Currently, while numerous studies have been focused on the technical and environmental feasibility of RA during their second life cycle based on laboratory control parameters, the critical issue is identifying and quantifying the environmental advantages of reusing and recycling in real scenarios. To achieve this goal, the Life Cycle Assessment (LCA) methodology proves to be a valuable tool. The holistic approach is crucial for decision-making in commercial production, as it evaluates economic, social, environmental, and quality aspects simultaneously (Dias et al. 2022; Tecco et al. 2016; Yu et al. 2023). There is a large amount of software LCA available in the market (Ben Abdallah et al. 2022). Notably, these tools integrate extensive databases related to life cycle inventories and impact assessment methodologies (Ben Abdallah et al. 2022). Specific software tools, such as SimaPro, Gabi, Umberto, JEMAI-LCA Pro, One Click LCA, and Open-LCA (De et al. 2017; D. Silva et al. 2017), assist users in conducting LCA-based studies. It is worth noting that each software has its unique characteristics, and results may vary depending on the chosen software (Lopes Silva et al. 2019; Speck et al. 2016).

Previous research works have used this free software applied on research studies of construction sector. Pamu et al. (2022) evaluated the environmental impact by using common building materials and alternate building materials. Besides, Smitha and Thomas (2022) analysed the life cycle impacts and circularity potential of materials used in the building searching for strategies promoting circularity.

## Experimental methodology

**Fig. 1:**
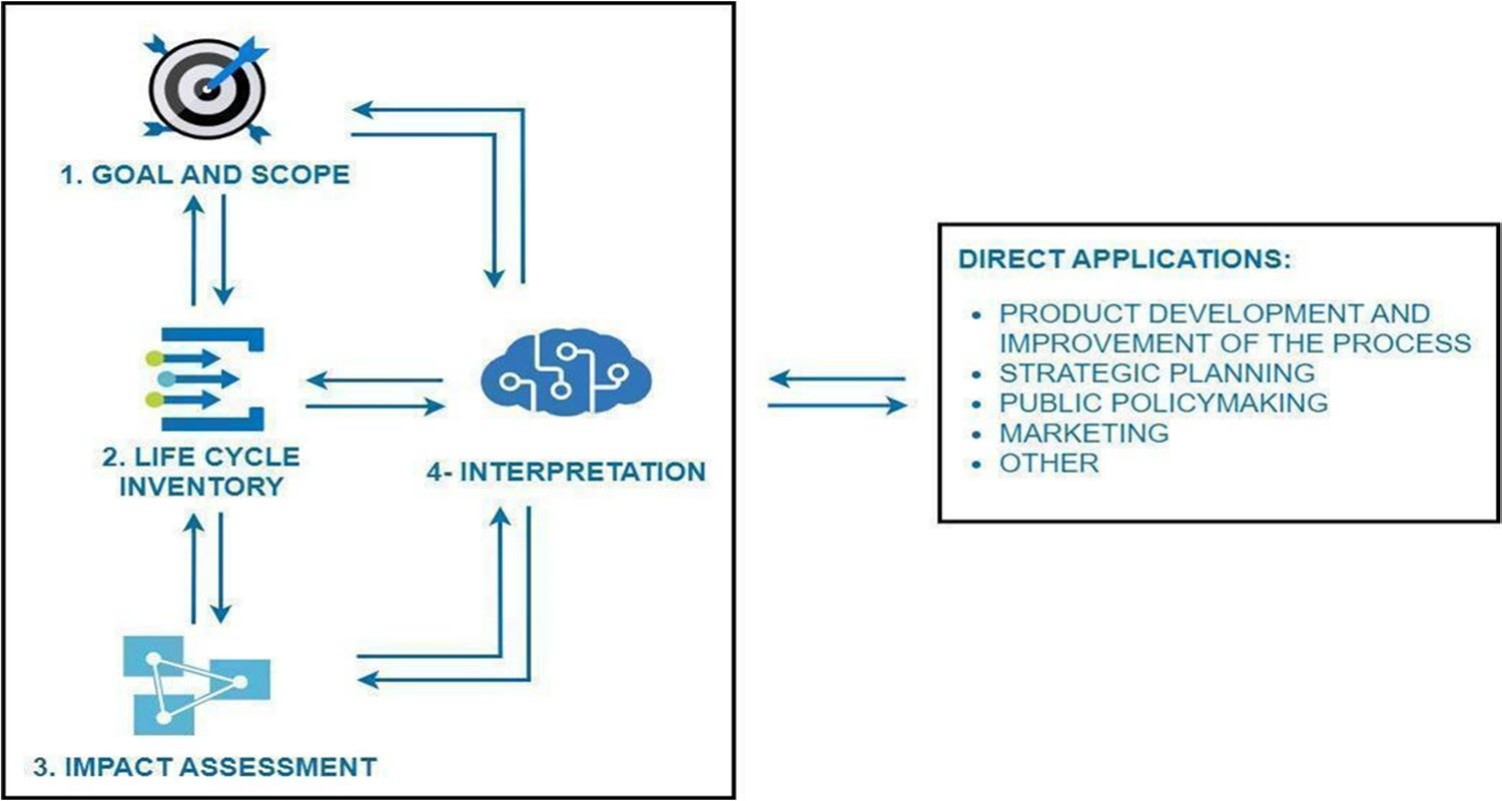
LCA methodological framework. Source: own elaboration based on UNE-EN-ISO 14040 (2021)

The present work developed the experimental methodology as described in Fig. 1 following the four main phases outlined in the standard ISO 14040: 2021:

1. Goal and Scope of the Study: In this initial phase, the subject of study is defined and justified. Additionally, the functional unit is determined.
2. Life Cycle Inventory: This phase encompasses data collection and calculation procedures to identify and quantify all adverse effects associated with the functional unit.
3. Impact Assessment: The results obtained from the inventory are utilized to assess the significance of the environmental impacts identified. This process entails associating inventory data with specific environmental impact categories, focusing solely on the environmental issues specified in the study's objectives and scope.
4. Interpretation: Finally, the results from the inventory are integrated with the impact assessment. This phase should yield results that are consistent with the defined objectives and scope, leading to conclusions, the identification of system limitations, and the provision of recommendations.

The goal of this work is to demonstrate that, in engineering applications, using recycled materials instead of natural aggregates is both technically and environmentally viable. To achieve it, the present work evaluates the advantages of using RA in construction applications for preserving natural resources, reducing CO2 emissions, lowering construction material costs, preserving land by reducing landfill use, and creating more employment opportunities in the recycling industry. The LCA methodology is applied to compare the environmental impacts of producing MRA from a CDW treatment plant and producing natural aggregates (NA) extracted from a quarry, both facilities located in the Province of Córdoba, Andalusia.

In our case study, it has been applied to product been development and improvement of the process, strategic planning, and marketing. The ECO LABEL certification programme (developed by Ecological Certification Institute) has been consulted, being designed to create sustainable solutions based on a life cycle assessment to reduce the environmental impact of product production activities. In the present study, which focused on LCA applied to construction materials, it was necessary to define three key aspects: (i) the functional unit, (ii) the system boundaries, and (iii) the impact categories.

### Functional unit

To enable comparisons among different products with similar functions, the LCA methodology employs a functional unit. This unit serves as the reference point against which all input and output flows of matter and energy within the system are normalized. It forms the fundamental calculation parameter for which all balances must be referenced. In this study, the functional unit is defined as one tonne of aggregate (for instance, when comparing two products designed to transport one ton of CDW from one location to another, the functional unit would be the transport of one ton per kilometre). Previous studies focused on the application of LCA methodology for environmental evaluation of aggregate production from recycled waste materials and virgin sources (Dias et al. 2022; Hossain et al. 2016) have applied the same functional unit since define overall objectives, system boundaries, and data sources of the studied production systems.

### System boundaries

The system boundaries established for most studies typically encompass the “cradle to gate” approach. This includes information modules A1, A2, and A3 (raw material supply, transportation, and product manufacturing, UNE-EN15804:2012 +A2:2020). This approach covers the minimum set of processes necessary for compliance. In the present study, the evaluation is focused on both production processes. For that, regarding the application of the LCA methodology in the case of recycled aggregates, it begins once the building is demolished. Specifically, the LCA system includes for (i) NA, the extraction of aggregates, the manufacture of raw materials, and their transportation, and for (ii) MRA, the transportation of CDW to the treatment plant, the processing of CDW in the studied plant to produce RA, and the transportation of RA to the customer.

### Impact categories

The selection of impact categories is crucial as it allows for result comparisons. In choosing these impact categories, we have considered ANNEX C of the UNE-EN 15804:2012+A2 Standard, which provides guidance on impact categories, associated parameters, methodologies, and characterization factors for all construction products and services. The selected impact categories are listed in Table 1.

**Table 1:** Environmental impact assessment categories in IMPACT 2002+

Impact categories	Damage category	Midpoint reference substance
Aquatic acidification, AQ	Ecosystem quality	kg SO2 eq
Aquatic ecotoxicity, EQ	Ecosystem quality	kg TEG water
Aquatic eutrophication, EUQ	Ecosystem quality	kg PO4 eq
Carcinogenic effects, EC	Human health	kg C2H3Cl eq
Global warming potential, GWP	Climate change	kg CO2 eq
Ionizing radiation, RION	Human health	Bq C-14 eq
Land occupation, LO	Ecosystem quality	m2org
Mineral extraction, ME	Resources	MJ surplus
Non-carcinogenic effects, ENC	Human health	kg C2H3Cl eq
Non-renewable energy, NRE	Resources	MJ primary
Ozone layer depletion, ODP	Human health	kg CFC-11 eq
Respiratory inorganics, RI	Human health	kgeq PM2.5 into air
Respiratory organics, RO	Human health / Ecosystem quality	kg C2H4 eq
Terrestrial acidification, AT	Ecosystem quality	kg SO2 eq
Terrestrial ecotoxicity, ET	Ecosystem quality	mol N eq

### Source of aggregates and processing characteristics

NA are mineral raw materials extracted from the earth and are used in various industrial sectors after undergoing crushing and classification processes. Different methods are employed for extraction operations, depending on the type of deposits. The two main exploitation systems are as follows:

1. Extraction using explosives (quarries): This method is used for compact stone deposits where explosives are necessary for material extraction. Subsequently, the extracted material is crushed and classified according to requirements.
2. Extraction using appropriate machinery and excavation technologies (gravel pits): Gravel pits are used to extract sediment, sand, and gravel from alluvial deposits. Since these materials are loose and not compact, mechanical extraction equipment is utilized to extract stone directly from the deposit. The extracted material is then classified into various sizes and compositions.

To establish the foundational inputs and outputs in the system, it is crucial to describe the production processes of the two construction materials studied in this work, MRA and NA.

Regarding MRA (an aggregate with a significant percentage of ceramic particles, exceeding 10% by weight), this study analyses the impact of producing one tonne of mixed recycled aggregates. This material is manufactured in a CDW plant associated with the Association of Construction and Demolition Waste Management Companies of Andalusia. The treatment process in the studied plant can be summarized as follows: (1) The process starts with the transportation of the CDW from the demolition site to the treatment plant. (2) At the plant, the material is prepared and cleaned, with different fractions being separated. CDW includes various components such as concrete, bricks, tiles, bituminous mixtures, gypsum, wood, glass, metals, plastic, solvents, asbestos, and soil. The non-hazardous inert fraction is the most abundant (Borghi et al. 2018). (3) The CDW is stored according to its classification, with stockpiles arranged in different areas based on their nature (e.g. concrete, mixed, asphaltic). (4) The CDW treatment has three stages (Barbudo et al. 2020). Pre-treatment reduces the volume of large elements using a demolishing or vibrating hammer. Primary treatment includes initial screening through a feed hopper, crushing the material, and performing a screening (separation) into variable sizes. Secondary treatment involves the use of an impact mill to provide the RA with a well-graded particle size distribution, classifying the aggregates into different commercial products. (5) The final phase in the plant is the quality control system, where the composition and physical properties of an RA are determined before its acceptance for use in new applications.

**Fig. 2:**
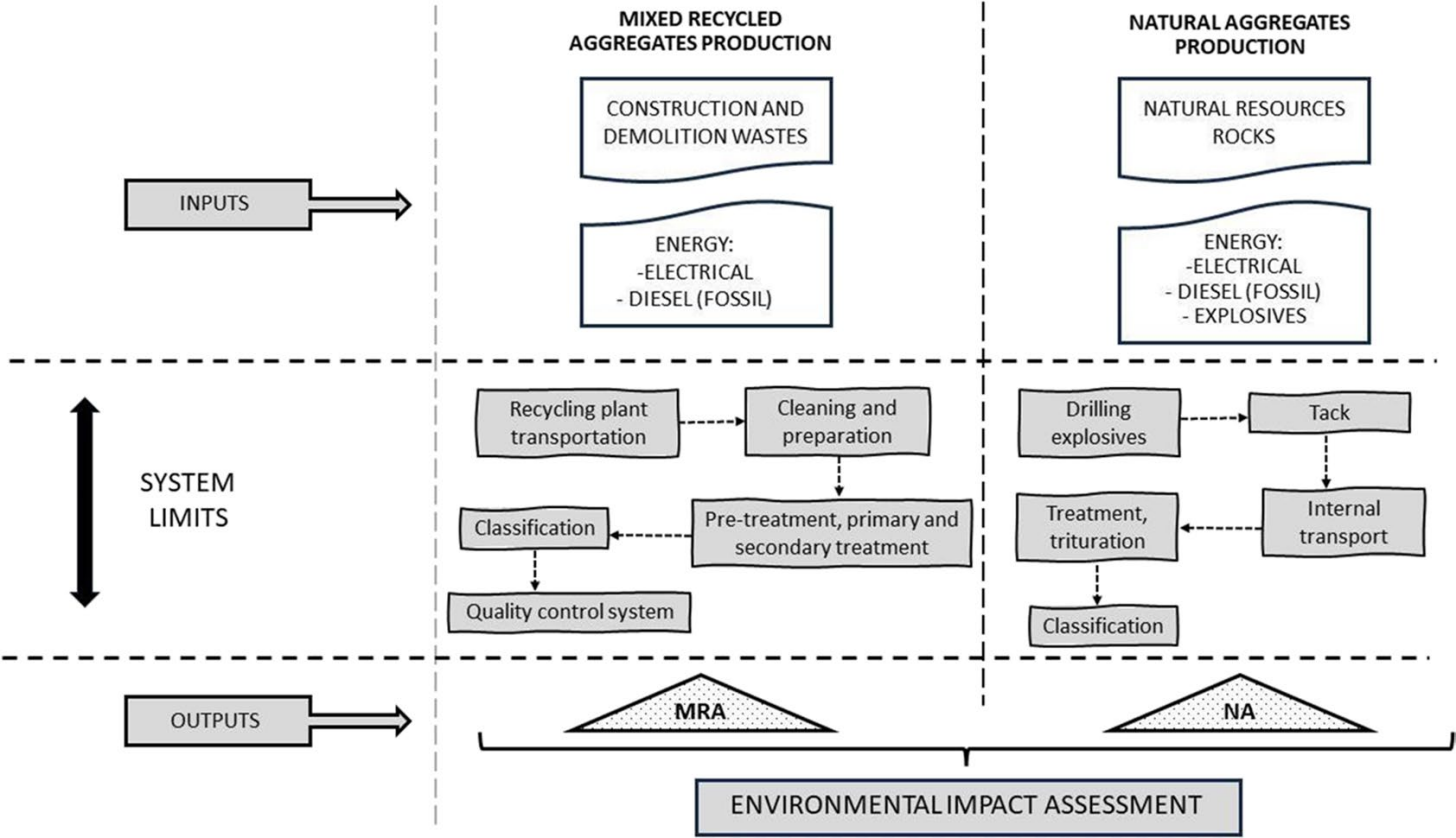
Scheme of production processes of NA in the quarry and RA in the treatment plant

As for NA, it comes from a quarry located in the province of Córdoba. The extraction process for this material can be summarized as follows: Limestones are typically mined using open-pit mining and underground mining methods, depending on economic and environmental conditions. The primary step in limestone mining involves breaking up the rock by detonating explosives placed in blast holes. A planned pattern of horizontal holes is drilled, with small holes outlining the desired block size. Explosives are then placed inside these holes to create a controlled blast that separates the rock from the bedrock (tacks). The extracted rocks are loaded onto haul trucks, which transport them to a rock crusher, often situated outside the quarry's mining area. Finally, the material is classified based on its grain size distribution. Both production processes are illustrated in Fig. 2 below.

According to the system described, the processes of both aggregates production (Fig. 2) are based on information provided by CDW plan managers and quarry operators operating in the province of Córdoba (Andalusia, Spain). The data collected for inventory generation introduced in OpenLCA software is presented in Table 2.

**Table 2:** Inventory data. Inputs to produce one tonne of MRA and one tonne of NA

Elements	Inputs for MRA production	Inputs for NA production
Water consumption	0.0058 m^3^/t	0.006 m^3^/t
Diesel consumption	0.3082 kg/t	0.34 kg/t
Electrical energy consumption	5.328 MJ/t	3.38 MJ/t
CDW	1.11 t	-
Transport of CDW from demolition site to plant	14 km/t	-
Explosives required per ton	-	0.18 kg/t
Transport of explosives from company to quarry	-	174 km/t
Internal transport of aggregates in the quarry	-	1 km/t

### Databases and source of datasets

In this study, various types of databases were considered. These included datasets containing specific data provided by CDW plant managers operating in the province of Córdoba (Andalusia, Spain), datasets from operators of the quarry where the NA was extracted (also located in Cordoba), and environmental product declarations (EPDs). Data that could not be supplied by the companies, recycling plants, and secondary sources were supplemented with existing databases, particularly the generic Ecoinvent database. Previous research (Pamu et al. 2022) has affirmed that the combination of the Ecoinvent database with the OpenLCA free software has consistently generated accurate results when compared to other software-database combinations. According to the present study, Open-LCA is the chosen software due to its capability to incorporate various databases and calculation models. This work utilizes Open-LCA because it is freely accessible to the entire scientific community.

### Environmental impacts and methods

The method used for evaluating the impacts was IMPACT 2002+, which developed by the Polytechnic University of Lausanne in Switzerland. his methodology is chosen because it best suits all selected impact categories. The IMPACT 2002+ life cycle impact assessment methodology employs a midpoint/damage approach that connects various types of life cycle inventory results (such as elementary flows and other interventions) through ten midpoint categories to four damage categories (Jolliet et al. 2003).

## Results and discussion

The economic and environmental impacts from the sources were discussed. To calculate the economic impacts, the production costs of recycled and natural aggregates were explained, as well as the final total cost for both products. Additionally, the environmental impacts were also included.

### Economic analysis of production costs

**Fig. 3:**
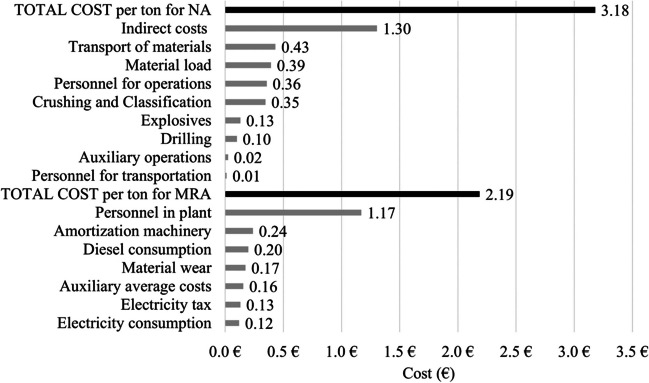
Production costs per ton of aggregate (NA and MRA)

The production costs per ton are depicted in Fig. 3. This cost assessment was conducted by consulting RA producers from CDW operating in the province of Córdoba and based on data from 2019. The analysis of cost is referred to 2021 as in 2022 and 2023, energy commodity prices experienced fluctuations, and significant deviations arose due to the fluctuating cost of energy and raw material prices, making it challenging for companies to provide representative average data.

Figure 3 displays the average values of production cost of 1 t of MRA in the province of Córdoba, calculated based on a daily production of one tonne, which is equivalent to 228 tonnes per year. The total cost amounts to 2.19 € per tonne of MRA. The cost data has been adjusted based on Spain annual general inflation rates (ref INE), obtaining a production cost of 2.27 €/t in 2021. In 2021, the average selling price for MRA in the province of Córdoba was 4.17 € per tonne (data obtained from the CDW treatment plants associated). As a result, the gross sales margin (excluding taxes) amounts to 45.56%.

For NA, production costs were obtained from a study carried out by Alfaro (2016). The production cost for 1 tonne of natural aggregate was 3.18 € per tonne. The cost data has been updated to account for an inflation rate of 6.9% from 2016 to 2021, resulting in a production cost of 3.40 € per tonne of NA. After consulting five quarries in the province of Córdoba, the average selling price of natural aggregates per tonne is 5.05 € per tonne, which is 21.10% higher than the price of MRA. This finding aligns with the research by Dias et al. (2022). Consequently, the gross sales margin (excluding taxes) for the sale of 1 tonne of natural aggregates reaches 32.67 %.

Based on this data, it is evident that MRA commands a lower selling price. It is widely well known that RA are a feasible alternative to NA for engineering and construction applications; it can be concluded that the use of mixed recycled aggregates in construction projects during their second life cycle is feasible. As a result, RA can remain competitive from an economic perspective, even when the transportation distances from the plant to the work sites exceed those of natural aggregates. This conclusion holds true without considering landfill taxes, which would further enhance the profitability of recycled aggregates. In Spain, these taxes are relatively low; for instance, in Madrid, this fee is approximately three € per cubic meter. In contrast, other European countries have varying landfill tax rates, ranging from three € per tonne in Italy to 63 € per tonne in Denmark (for inert waste) (Luciano et al. 2022).

The present section analyses the economic properties with various aggregate types as the study holistic approach for valorization of recycled aggregates. Previous studies corroborate the data obtained since previous authors have meticulously evaluated not only the mechanical and durability data, but also the environmental and economic properties. As an example applied to Lightweight Self-Compacting Mortars manufactured with various aggregate types, Ulas et al. (2024) present a study with economic aspect in recycled concrete. Likewise, analysing the cost differences, it has also been observed that the production of concrete with recycled aggregates is more economical than the production of concrete with natural aggregates (Ulucan and Alyamac 2022)

### Evaluation of the impacts generated by the productive processes of MRA and NA

The environmental impact resulting from the exploitation of natural resources and the excessive consumption of land for landfills drives the need to increase the application of RA. Previous studies (Del Rey et al. 2018; Galvín et al. 2018; López-Uceda et al. 2019) have identified the key components of RA that pose environmental risks and have extensively demonstrated the feasibility of their use in various engineering applications. The technical feasibility of RA is directly linked to the treatment system, as proper operations result in higher-quality aggregates that are highly competitive with natural aggregates (Barbudo et al. 2020). In this context, this section analyses the environmental impact generated by the treatment process of RA and the extraction process of NA.

The life cycle considered for MRA includes the raw materials necessary for its production, their transportation from the demolition site to the production plant, the processing of the aggregates, and the final production of the material for engineering applications. Consequently, the transportation to the site-work where it will be applied in its second life cycle has not been considered, nor have aspects such as conservation, maintenance, and waste management at the end of the useful life of the materials (see Fig. 2). In the case of NA, the life cycle considered encompasses the raw materials required for aggregate extraction from the quarry (including explosives and their acquisition and transportation to the quarry), as well as the processing and production of the material (energy consumption, equipment, etc.). Transport of NA to the site work after manufacturing has not been considered either.

Based on the systems defined in Fig. 2 and the impact categories listed in Table 1, the contributions of each process to different impact categories for each material, MRA and NA, are summarized in Tables 3 and 4.

**Table 3:** Contribution of each process to the different impact categories in MRA production

Impact category	Reference unit	MRA process	Diesel	Water	Electricity	Transport to plant
AQ	kg SO2 eq	*ni*	0.0007	7.55E−06	0.00588	0.0087
EQ	kg TEG water	*ni*	19.965	0.056	25.983	4.109
EUQ	kg PO4 P-lim	*ni*	2.754E^−06^	1.28E^−06^	2.91E^−06^	1.11E^−05^
EC	kg C2H3Cl eq	*ni*	5.89E^−05^	1.46E^−06^	0.0003	0.0004
GWP	kg CO2 eq	*ni*	0.083	0.003	0.788	1.351
RION	Bq C-14 eq	*ni*	0.114	0.003	16.765	0.322
LO	m2org.arable	*ni*	0.041	*ni*	*ni*	*ni*
ME	MJ surplus	*ni*	1.00E^−06^	*ni*	6.13E^−06^	3.39E^−07^
ENC	kg C2H3Cl eq	*ni*	0.008	5.58E^−06^	0.002	0.001
ODP	kg CFC-11 eq	*ni*	2.98E^−10^	3.39E^−11^	1.65E^−07^	*ni*
RI	kg PM2.5 eq	0.0007	7.66E^−05^	1.00E^−06^	0.0007	0.0017
RO	kg C2H4 eq	*ni*	7.53E^−05^	1.01E^−07^	8.72E^−05^	0.0004
AT	kg SO2 eq	*ni*	0.0022	2.73E^−05^	0.0117	0.0503
ET	kg TEG soil	*ni*	15.830	*ni*	0.492	0.2803

*ni* null impact

**Table 4:** Contribution of each process to the different impact categories in NA production

Impact category	Reference unit	NA Process	Explosive production	Internal transport	Diesel	Electricity	Explosives transport
AQ	kg SO2 eq	0.047	0.0012	0.0003	0.0011	0.0045	1.29E−05
EQ	kg TEG water	10113.415	8.386	0.132	27.928	17.781	0.005
EUQ	kg PO4 P-lim	*ni*	*ni*	5.23E^−07^	3.90E^−06^	5.01E^−06^	2.16E^−08^
EC	kg C2H3Cl eq	*ni*	0.002	1.46E^−05^	8.26E^−05^	0.0002	6.07E^−07^
GWP	kg CO2 eq	0.016	0.216	0.0490	0.1320	0.6077	0.0020
RION	Bq C-14 eq	*ni*	1.209	0.012	0.182	13.074	*ni*
LO	m2org.arable	*ni*	0.001	*ni*	0.064	*ni*	*ni*
ME	MJ surplus	*ni*	0.013	1.07E^−08^	1.50E^−06^	4.11E^−06^	4.43E^−10^
ENC	kg C2H3Cl eq	0.010	0.002	2.98E^−05^	0.0125	0.0013	1.25E^−06^
NRE	MJ primary	*ni*	3.617	*ni*	*ni*	*ni*	*ni*
ODP	kg CFC-11 eq	*ni*	2.55E^−08^	1.07E^−10^	4.38E^−10^	1.18E^−07^	4.42E^−12^
RI	kg PM2.5 eq	0.008	0.0002	5.85E^−05^	0.0001	0.0005	2.59E^−06^
RO	kg C2H4 eq	*ni*	3.21E^−05^	1.47E^−05^	0.0001	6.12E^−05^	6.94E^−07^
AT	kg SO2 eq	0.367	0.007	0.002	0.004	0.011	9.38E^−05^
ET	kg TEG soil	2593.049	2.502	0.012	28.935	0.441	0.0005

*ni* null impact

Table 3 displays the impact contributions of the various processes involved in the production of 1 tonne of mixed recycled aggregate. (Non-renewable energy, NRE, is not included due to the negligible impact obtained.) The detailed description of each process considered in the impact calculation is as follows:

1. MRA process: It is equivalent to the production of one tonne of MRA, which affects mainly dust emissions.
2. Diesel: This process corresponds to diesel production at a refinery, involving a production mix from crude oil and/or biocomponents.
3. Water: It refers to drinking water, production mix, at the plant, water purification treatment, and/or groundwater usage.
4. Electricity: It corresponds to electricity mix and/or consumption mix, at consumer level, AC, 115-220V.
5. Transport to plant: It involves the use of a lorry with a total weight of 22 tonnes and a maximum payload of 17 tonnes as a reference for transporting CDW from the demolition site to the treatment plant.

Table 4 summarizes the impact contributions of the different processes involved in the production of one tonne of NA, as follows:

1. NA process: This represents the environmental contribution equivalent to the production of 1 tonne of NA extracted from the quarry.
2. Explosive production: It refers to the production of explosives composed of ammonium nitrate (33.5% N) at the plant.
3. Internal transport: This involves the use of an articulated lorry for internal transport of aggregates within the quarry. The lorry has a total weight of 40 tonnes and a maximum payload of 27 tonnes.
4. Diesel: This process corresponds to diesel production at a refinery, involving a production mix from crude oil and/or biocomponents
5. Electricity: This corresponds to the electricity mix and/or consumption mix at the consumer level, AC, 115-220V.
6. Explosive transport: This involves the use of a lorry (with a total weight of 22 tonnes and a maximum payload of 17.3 tonnes) to transport explosives from the supplier to the quarry.

**Fig. 4:**
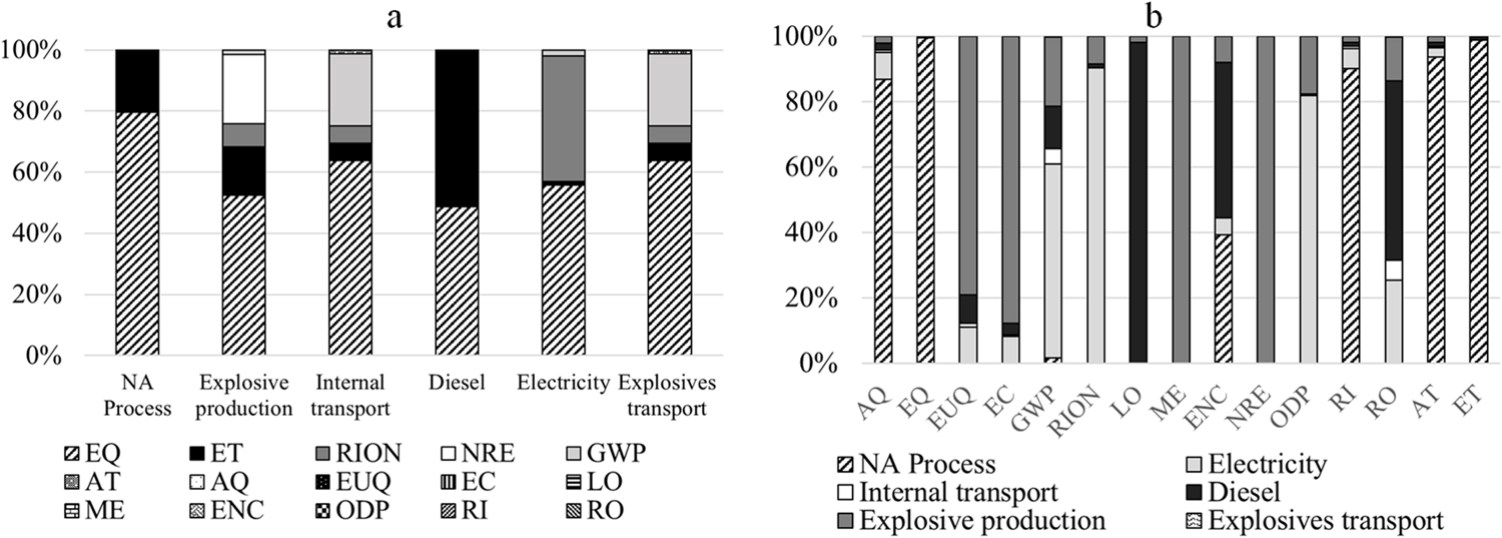
Impact contribution of NA production. **a** By processes. **b** By impact categories

To discuss the results and highlight the differences between the evaluated processes, Figure 4 represents the contribution values of each production process of NA for each impact category (as listed in Table 1) on a relative scale, ranging from 0% to 100%.

As depicted in Fig. 4, the impact categories most significantly affected by the various processes involved in NA production are as follows: aquatic ecotoxicity (EQ), terrestrial ecotoxicity (ET), ionizing radiation (RION), non-renewable energy (NRE), and global warming potential (GWP). The NA production process exerts the most substantial impact on aquatic ecotoxicity, accounting for 10,113.415 kg TEG water (10.113 tonne TEG water), and on terrestrial ecotoxicity, resulting in 2,593.04 kg TEG soil (2.597 tonne TEG water). These high values stem from the aggressive nature of extracting aggregate from the ground. Explosive production also contributes to aquatic ecotoxicity with an impact of 8.38 kg TEG water and non-renewable energy with 3.61 MJ primary.

Concerning the considered transport systems, as expected, both have a more significant impact on global warming potential: Internal transport contributes 0.048 kg CO_2_ eq, while explosive transport contributes 0.002 kg CO_2_ eq. Additionally, they affect the aquatic ecotoxicity category with 0.13 kg TEG water and 0.005 kg TEG water, respectively. Lastly, the diesel production process significantly impacts the aquatic ecotoxicity category with 27.92 kg TEG and terrestrial ecotoxicity with 28.93 kg TEG soil. Meanwhile, electricity consumption contributes 17.78 kg TEG to aquatic ecotoxicity and 13.07 Bq C-14 eq to ionizing radiation. Therefore, it is evident that the manufacturing process of 1 tonne of NA from a quarry exerts the greatest impact on the aquatic ecotoxicity category (Rosado et al. 2017). Consequently, it can be concluded that during NA production, the most affected processes is diesel, which primarily influences the impact categories LO and RO; electricity, which primarily influences the impact categories RION and ODP, and explosive production, which primarily influences ME and ODP.

**Fig. 5:**
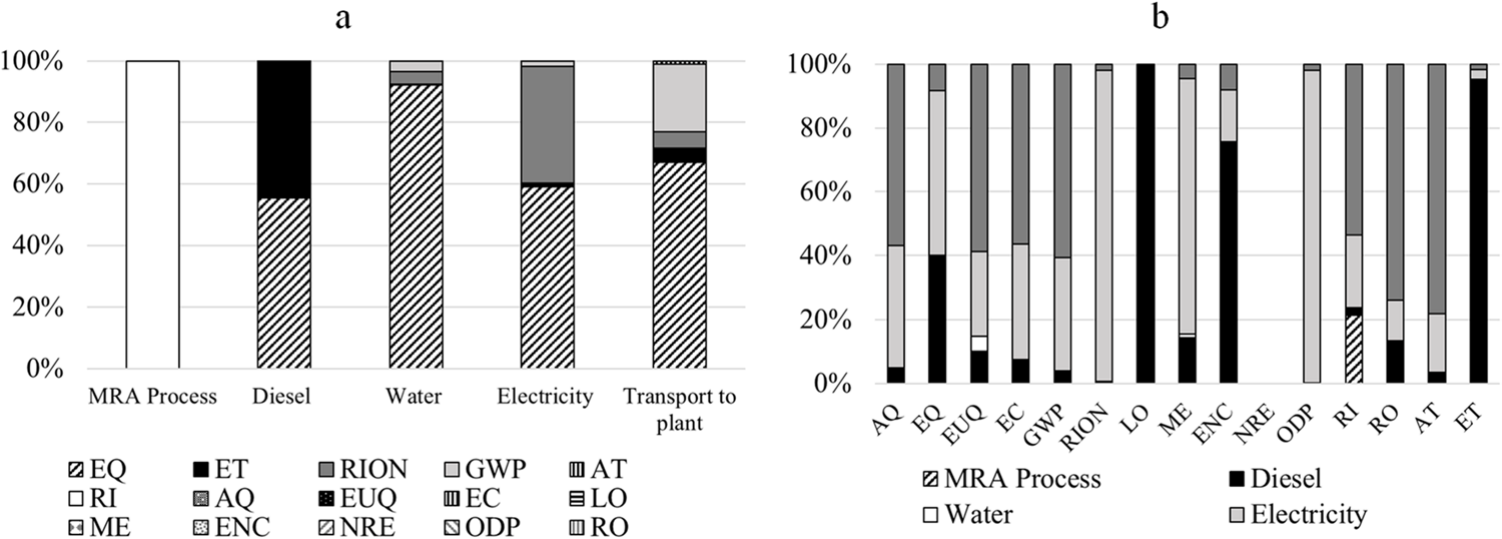
Impact contribution of MRA production. **a** By processes. **b** By impact categories

From Fig. 5, it can be identified the most affected impact categories in the production of 1 tonne of MRA at the CDW treatment plant: EQ, ET, RION, and GWP. The MRA treatment process primarily impacts respiratory inorganics, contributing only 0.0008 kg PM2.5 eq. This impact arises because the process focuses solely on transforming CDW into RA, with crushing being the most significant operation, generating suspended dust emissions into the atmosphere. During treatment at the plant, diesel consumption leads to an impact of 20.37 kg TEG on the EQ category and 28.93 kg TEG on ET. Additionally, electricity consumption results in a 26.51 kg TEG impact on EQ and a 19.49 Bq C-14 eq impact on RION. Finally, water consumption does not exhibit significant values but has the highest impact on EQ, contributing 0.077 kg TEG water.

Consequently, it can be concluded that during MRA production, the most affected processes are diesel, which primarily influences the impact categories LO and ET; electricity, which primarily influences the impact category ODP; transport to plant, which primarily influences the impact categories RO and AT; and, finally, container, which affects all impact categories as depicted in Fig. 5. Regarding to the influence of transport on the environmental impact, previous works of Hossain et al. (2016) have confirmed that the variations between both the production process (natural and recycled aggregates) are largely due to different transports and distances which are consistent with the obtained data by the present study.

### Comparison between the environmental impact generated by natural and recycled aggregates

**Fig. 6:**
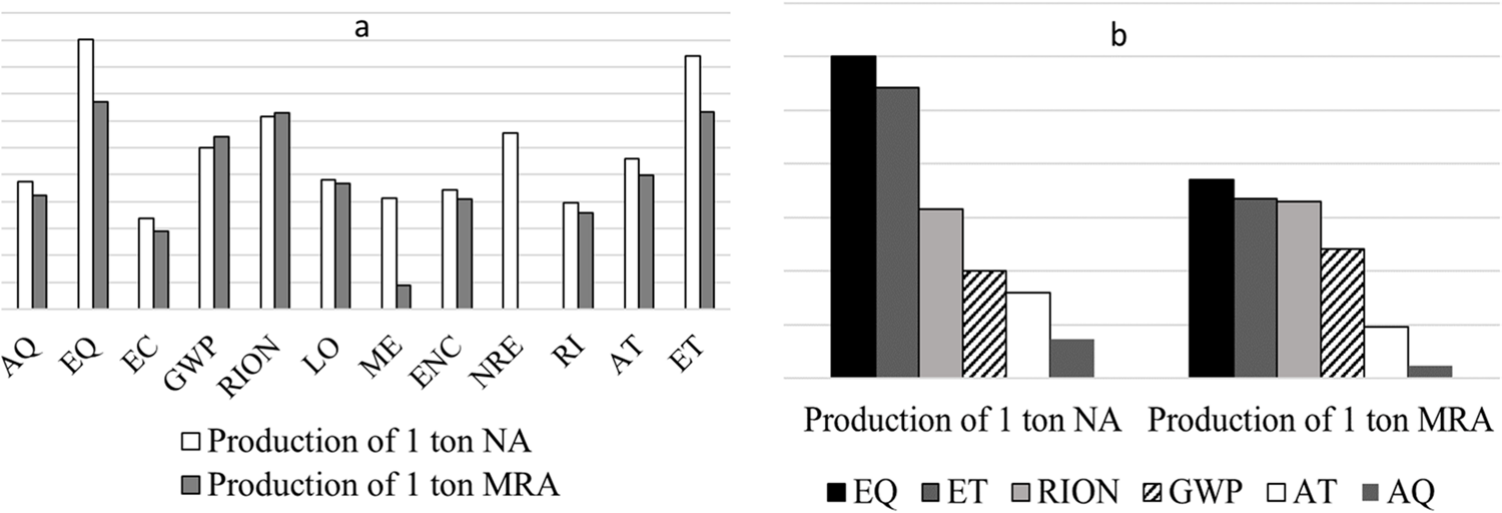
Comparative of impacts generated in the production of NA and MRA

To establish a comparison between both production processes, Table 5 and Fig. 6 compare the total impact generated by the production of each material. This section includes the total cumulative values of the MRA and NA processes, which correspond to the sums of the individual values of the total MRA process (composed of five processes: MRA process, diesel, water, electricity, transport to plant) and NA (composed of six processes: NA process, explosive production, internal transport, diesel, electricity, explosives transport) based on the system boundaries and processing characteristics described in Section "Experimental methodology".

**Table 5:** Comparison between the environmental impact generated by MRA and NA process

Impact category	Reference unit	Total of MRA process	Total of NA process
AQ	kg SO2 eq	0.015	0.053
EQ	kg TEG water	50.133	10167.647
EUQ	kg PO4 P-lim	*ni*	*ni*
EC	kg C2H3Cl eq	8.0E^−04^	0.002
GWP	kg CO2 eq	2.226	1.023
RION	Bq C-14 eq	17.206	14.477
LO	m2org.arable	0.041	0.065
ME	MJ surplus	6.97E^−02^	0.013
ENC	kg C2H3Cl eq	0.0115	0.026
NRE	MJ primary	*ni*	3.617
ODP	kg CFC-11 eq	*ni*	*ni*
RI	kg PM2.5 eq	0.003	0.009
RO	kg C2H4 eq	0.0005	0.001
AT	kg SO2 eq	0.0644	0.391
ET	kg TEG soil	16.603	2624.937

*ni* null impact

When comparing the environmental impact generated by the production of 1 tonne of each construction material (refer to Table 5), the following deductions can be made. For both NA and MRA, the most affected categories are EQ (10,167.64 and 51.15 kg TEG water, respectively) and ET (2,624.93 and 22.13 kg TEG soil, respectively). In the third and fourth positions, the categories with the greatest environmental impact are RION (14.47 and 20 Bq C-14 eq, respectively) and GWP (1.02 and 2.55 kg CO2 eq, respectively).

According to the data represented in Fig. 6 (data expressed in the reference units shown in Table 5), notable differences in the levels detected in each construction material can be observed despite both productive processes, MRA and NA, having the most affected impact categories as EQ and ET (see weighted data in Fig. 6b). The values of environmental damage are significantly higher during NA production compared to the MRA production process, as clearly shown in Fig. 6a. In terms of EQ, NA production results in an impact almost 200 times higher than that of the MRA production process. Similarly, ET results are 120 times higher in NA production compared to MRA. On RION, it shows similar values in both cases. On the other hand, GWP values are 2.5 times higher in the case of MRA compared to NA, primarily due to emissions during the transport of CDW from the demolition site to the treatment plant, resulting in an impact of 1.55 kg CO_2_ eq.

Therefore, based on the analysed data, it can be concluded that the accumulated impact values generated during the manufacturing of one tonne of NA are significantly greater than those produced during the production of one tonne of MRA, confirming the environmental benefits of this recycled materials compared to the extraction of natural materials. This statement has been corroborated by other studies focused on comparison of production processes between natural and recycled aggregates. In this case, the LCA results showed that about 49–51% net environmental impacts can be reduced during the production of recycled aggregates from CDW instead of producing aggregates from crushed stone (Hossain et al. 2016). The data studied lead us to the conclusion of the feasibility of using RAs as an alternative to the exploitation of natural resources. Nevertheless, there is a lack of research papers that delve into the application of the LCA methodology applied to RAs and NAs simultaneously (Dias et al. 2022).

## Conclusions

According to the results, it can be deduced that most of the impacts are attributed to the acquisition of raw materials: explosives in the case of natural aggregates and the transportation of Global Warming Potential to the treatment plant in the case of recycled materials, as well as the diesel used in the treatment plants. Regarding the production of one tonne of mixed recycled aggregates, it is significantly more beneficial than the production of one of NA, with a lower impact in most of the evaluated categories, particularly in terms of aquatic acidification, aquatic ecotoxicity, ionizing radiation, and global warming.

In terms of Aquatic Ecotoxicity, NA production results in an impact almost 200 times higher than that of the mixed recycled aggregates production process. Similarly, Terrestrial Ecotoxicity results are 120 times higher in natural aggregates production compared to mixed recycled aggregates. Ionizing radiation, it shows similar values in both cases. On the other hand, Global Warming Potential values are 2.5 times higher in the case of mixed recycled aggregates compared to natural aggregates, primarily due to emissions during the transport of construction and demolition waste from the demolition site to the treatment plant, resulting in an impact of 1.55 kg CO_2_ eq.

Besides the environmental aspect, the economic analysis conducted in this study demonstrates that it is also more economically advantageous since the cost of producing recycled aggregates is over 30% cheaper than natural aggregates, being more competitive even when the transportation distances from the plant to the work sites exceed those of natural aggregates. Therefore, the present study contributes to quantifying the environmental and economic benefits of promoting a circular economy in the construction sector by maximizing recycling ratios, ensuring the sustainability of a sector that is currently under global scrutiny.
